# Cardiovascular Disease Care Beyond the Cardiologist: An Overview of the Rollout of Transthoracic Echocardiography Training and Services in Kenya

**DOI:** 10.5334/gh.1437

**Published:** 2025-05-30

**Authors:** Daniel Muriuki, Marietta Ambrose, Hassan Ahmed, Michael Foster, Hellen Nguchu, Lee Goldberg, Bernard Samia

**Affiliations:** 1 Meru Teaching and Referral Hospital, KE; 2 Holo Global Health Research Institute, KE; 3 Faculty, KCS-ACC Echocardiography Training Program, KE; 4 University of Pennsylvania, US; 5 Kenyatta Teaching and Referral Hospital, KE; 6 Moi Teaching and Referral Hospital, KE; 7 Mwai Kibaki Teaching and Referral Hospital, KE; 8 MP Shah Hospital, KE; 9 Kenya Cardiac Society, KE

**Keywords:** Transthoracic Echocardiography Training, Kenya Cardiac Society (KCS), American College of Cardiology (ACC), Cardiac Imaging in Resource Limited Settings, Tasksharing essential cardiology services with non-cardiologists

## Abstract

Cardiovascular diseases (CVDs) are a leading cause of mortality in low- and middle-income countries (LMICs), yet access to echocardiography remains limited due to workforce shortages. The Kenya Cardiac Society (KCS), in collaboration with the American College of Cardiology (ACC), launched a 16-week transthoracic echocardiography (TTE) training program to address this gap. This blended learning initiative trains non-cardiologist healthcare workers through online modules, hands-on workshops, and expert mentorship. Since 2022, the program has trained 95 participants, enhancing diagnostic capacity and expanding echocardiography services to underserved areas. Early outcomes include reduced patient travel distances, improved early detection of cardiac conditions, and strengthened CVD management at secondary and tertiary levels. Challenges such as limited equipment access and financial constraints persist, but strategic partnerships and innovative training models demonstrate the program’s potential for scalability. The KCS-ACC-TTE program highlights the effectiveness of task-sharing and collaboration in strengthening cardiovascular care, offering a replicable framework for LMICs to improve access to essential cardiac diagnostics.

## Background

Cardiovascular disease (CVD) is the world’s leading cause of death, with more than 20 million people dying annually (1). The CVD burden is disproportionately higher in low-to-middle-income countries (LMICs) in Sub-Saharan Africa including Kenya (2,3,4,5). LMICs need to adopt robust and sustainable secondary and tertiary prevention and management strategies to minimize the devastating effects of CVD (1).

Transthoracic echocardiography (TTE) is an essential tool that provides non-invasive cardiac imaging for the effective diagnosis and guidance of cardiovascular conditions (6,7). It has a high diagnostic value in resource-limited settings where access to specialists and multi-modality imaging technology is limited (8,9,10,11). A TTE is used to evaluate cardiac structure and function and diagnose cardiomyopathies, valvular heart disease, congenital heart disease, pericardial disease, and right heart disease, among others (8). When combined with Lung Ultrasound (LUS), it can increase the sensitivity of heart failure diagnosis in LMICs where cardiac biomarkers such as brain natriuretic peptides (BNP) are costly and inaccessible (12).

Echocardiography and adjunct sonographic modalities like lung ultrasound are easily applicable in ambulatory, in-patient, emergency, and critical care settings (13). The widespread adoption of portable equipment in secondary and tertiary public facilities in LMICs has enabled cardiac ultrasound to be offered at the point-of-care (10,14,15). In LMICs, non-experts can receive focused echocardiography training to improve access to cardiac diagnostic services and augment efforts in managing the rise of CVD (9,10,16,17). However, the provision of echocardiography services has traditionally been the work of the few available cardiology experts, which has limited patient access to cardiac diagnostics in LMICs (9,11). Task sharing echocardiography services with non-cardiologists may improve the human resources available to provide cardiac diagnostics in resource-limited settings (27). We review an ongoing innovative and collaborative transthoracic echocardiography capacity-building training program in Kenya targeted at non-cardiologists to enhance cardiac diagnostics and CVD care.

### Resources for CVD care provision in Kenya

To combat its rising CVD burden, Kenya is rolling out the WHO HEARTS technical package as outlined in the 2024 edition of the Kenya National CVD management guidelines (6). Primary care is provided at level 1, level 2, and 3 healthcare facilities. Level 1 and 2 facilities are tasked with health promotion and screening for CVD, while level 3 facilities are also tasked with managing uncomplicated disease. Human resources available at these lower levels of care include Community Health Workers (CHWs), nurses, clinical officers, and medical officers. Basic diagnostic equipment for CVD care at level 3 facilities includes radiography and electrocardiography machines (6).

Secondary and tertiary care consists of level 4, level 5, and 6 hospitals. Level 4 facilities provide comprehensive diagnosis and care of complicated CVD by medical doctors and internal medicine physicians. Advanced multi-disciplinary level care of more complex CVD patients, rehabilitation, and follow-up is done at level 5 and 6 facilities. Human resources available at these latter levels include health care workers (HCWs) at levels 2 and 3 facilities, internal medicine physicians, cardiologists, cardiovascular surgeons, and other technical support staff like echocardiographers and cardiac perfusionists. Equipment for basic and advanced CVD diagnosis, drugs for guideline-directed medical therapy, and infrastructure for operative or invasive interventions are also available at these higher-level facilities (6).

**Figure 1:**
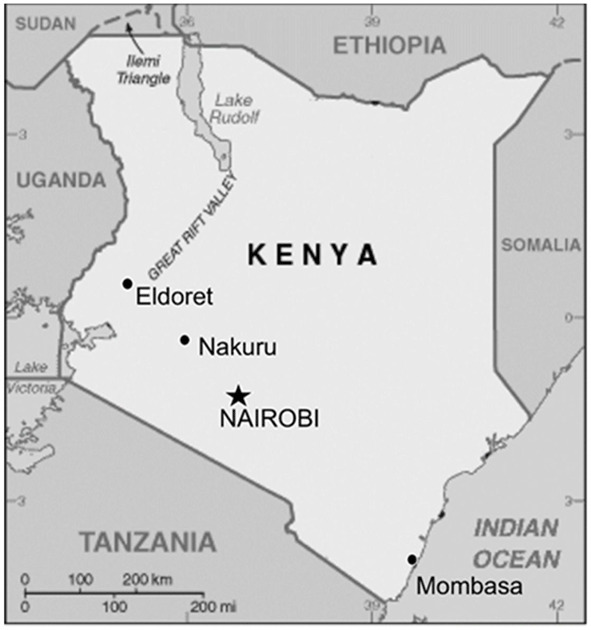
A map of Kenya showing the geographic localization of cardiology experts in 2009. No current data mapping their geographic distribution is available. (Courtesy, Binnanay et al. (16)).

Only 18% of Kenya’s healthcare facilities provide specialized cardiac services (18). While the 2024 Kenya National CVD Guidelines list echocardiography as an essential cardiology service, such services are limited to only a few tertiary level 5 and level 6 facilities. The exact number of facilities offering this service and the nature of the service provided is undocumented. Performing a standard echocardiogram is traditionally reserved for cardiologists and cardiac technologists, but this poses a challenge in Kenya since such specialists are scarce (9,19)(See Figure 1 below).

Kenya has about 40 cardiologists, 350 internal medicine physicians, 7,500 medical officers, and 25,000 clinical officers (20). These personnel serve about 30 million adults (3,21). The doctor-patient ratio in Kenya is 1.6 per 10,000 of the population, comparable to Africa’s average of 2.0 per 10,000. Most Kenyan CVD specialists work in the private sector, and in urban centers (19,21,22), yet the 2022 Kenya Demographic and Health Survey (KDHS) recorded 70% of Kenya’s population living in rural areas (21). Rural areas are therefore generally underserved and patients often require referral to few tertiary or private facilities to access echocardiography and other specialist cardiology services.

Specialized CVD training programs have emerged to bridge this human resource gap. These include cardiology fellowship training programs by The Aga Khan University Hospital, The Moi University, and cardiology diplomas for clinicians offered by The Karen Hospital, and other providers (19,23). These institutions graduate a limited number of specialist cardiologists, cardiology clinical officers, and cardiac technologists annually.

The inequity in distribution and access to human resources for CVD care creates a case for brief, high-impact training on high-yield interventions such as echocardiography to improve access. Non-cardiologists (including internal medicine physicians and medical, clinical, and nursing officers) who form the majority of HCWS in rural communities, secondary and tertiary facilities are ideal candidates to target for capacity building in echocardiography. The effectiveness of such tailor-made training programs is supported by increasing evidence that specific competencies in ultrasound applications can be attained and retained by non-specialists after a brief training (24). In Kenya, training midwives in point-of-care obstetric ultrasound resulted in acceptable knowledge retention, positively impacting routine clinical decision-making in the care of pregnant women (25). Rural HCWSs could competently perform and interpret obstetrics and extended focused abdominal ultrasounds for trauma (E-FAST) using hand-held devices after sensitization training (26). The evidence generated from these training experiences is encouraging and can be extrapolated to the training of non-cardiologists in cardiac ultrasound applications in LMICs.

### A case for transthoracic echocardiography capacity building for non-experts in Kenya

Nurses can utilize basic echocardiography to diagnose cardiac disease and form a front-line workforce in a nurse-led heart failure program in Rwanda’s district hospitals (10). Eighty four percent of medical trainees in Kenya with no prior experience in ultrasound use competently diagnosed the five most common causes of dyspnea of cardiac origin after a 23-hour training in focused cardiac ultrasound (9,16). However, the quality of training, supervision and practice of cardiac ultrasound applications affects competency (9). Non-experts can, therefore, acquire basic cardiac ultrasound skills after adequate training and supervision (24). This evidence supports training non-experts in echocardiography to facilitate task sharing.

Task sharing occurs through the collaborative performance of functions by HCWSs at various levels of expertise (27). Effective training of non-expert HCWSs in LMICs can determine the successful implementation of task sharing in managing CVD (2). However, effective task sharing also requires high quality training programs, mentorship during and after training, and regulatory oversight to ensure ethical practices (28).

Task sharing may be replicated in capacity-building non-experts in echocardiography skills in LMICs to improve CVD care at the secondary and tertiary levels of care. This could improve service provision especially in rural areas in Kenya which have a disproportionately higher CVD burden, a need for more human resource for CVD care, and a lack of other non-personnel resources for CVD care. Supervised training of HCWSs such as internal medicine physicians, medical officers, nurses, and clinical officers in level 1 echocardiography skills could bridge the critical gap in access to cardiac diagnostics and care faced by most Kenyans. To achieve this, the Kenyan Cardiac Society (KCS) has collaborated with the American College of Cardiology (ACC) to train Transthoracic Echocardiography (TTE) to these HCWSs. Enabling these HCWSs to perform echocardiography will improve the human resource available for CVD diagnosis and care in Kenya and improve access to these services.

### The Kenya Cardiac Society-American College of Cardiology Transthoracic echocardiography (KCS-ACC-TTE) training program

**Figure 2:**
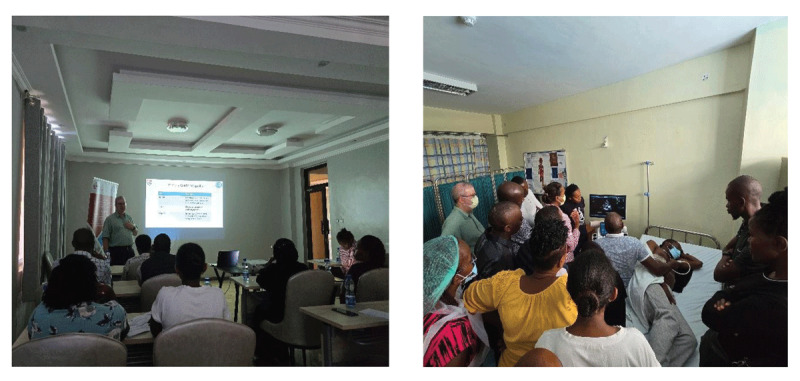
In-person (left) and hands-on training (right) sessions in January 2024 with KCS-ACC faculty. Photo: Mwai Kibaki Teaching and Referral Hospital (MKTRH). reproduced with permission from MKTRH.

Established in 2022, the KCS-ACC-TTE blended training program is delivered over 16 weeks. Training entails a series of interactive, live, online sessions with lecturers teaching the basics of cardiac anatomy and physiology, echocardiographic chamber quantification, valve, and hemodynamic assessment, and an introduction to advanced echocardiography modalities. The training curriculum covers cardiac pathologies, including cardiomyopathies, valvular heart disease, ischemic heart disease, diseases of the right heart, pericardial disease, congenital heart disease, point-of-care and critical care echocardiography, and indications for multi-modality imaging. Two to three in-person practical workshops are conducted during the 16 weeks. These workshops run over three consecutive days at tertiary centers with a high volume of CVD patients. Workshops entail brief in-person sessions on specific pre-determined thematic areas in echocardiography, such as valve assessment, chamber quantification, hemodynamics, and specific cardiac disorders of interest, including cardiomyopathy, valvular heart disease, right heart diseases, or congenital heart diseases. During the workshops, trainees also get exposure and practice of hands-on imaging skills on cardiac patients with varied pathology under expert faculty supervision (See Figure 2).

The faculty comprises Kenyan specialists and visiting experts from the ACC, which enriches the course with a quality blend of locally curated learning material, evidence, and experience drawn from global centers of excellence in cardiac care. Trainees take written pre- and post-course tests at the beginning and end of the training program, respectively, and are certified upon attaining 70% as the pass mark. During the course, students also receive logbooks mandating a minimum of 70 echocardiography examinations, which must be performed under supervision of patients with various pathologies in a hospital setting. The faculty also supports participants through a WhatsApp™ discussion forum, where additional learning materials are shared and challenging cases reviewed. At the end of the course, the participants are expected to be competent in basic TTE image acquisition of parasternal, apical, substernal and suprasternal views; in level 1 chamber quantification using 2D, M-mode, and doppler for valve, tissue velocity and hemodynamic assessments; and in interpreting common echo findings including but not limited to a normal heart, hypertensive heart disease, heart failure phenotypes, valvular heart disease, cardiomyopathies, complications of acute coronary syndromes, ischemic heart disease, pulmonary hypertension, right heart failure, pericardial effusion and other pericardial diseases, and common congenital heart diseases such as septal defects.

**Figure 3:**
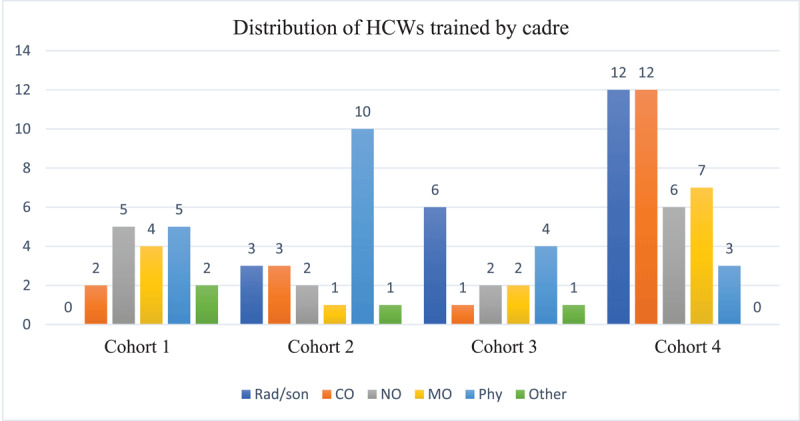
Distribution of HCWs by cadre across the four training cohorts. Key: Rad/son- Radiographer/sonographer, CO- Clinical Officer, NO- Nursing Officer, MO- Medical Officer, Phy- Physician.

Since its inception, the KCS-ACC-TTE program has successfully trained 95 participants in four cohorts. Participants trained consist of internal and family medicine physicians (23%; n = 22), radio-sonographers (22%; n = 21), clinical officers (20%; n = 19), nursing officers (16%; n = 14), medical officers (15%; n = 14), and other HCWSs (4%; n = 4) including 2 anesthesiologists, 1 nephrologist, and 1 cardiothoracic surgeon. A description of the distribution of trainees for each cohort is illustrated in Figure 3.

This training costs about $1,400 per participant, with expenses incurred mainly on logistical support during the physical in-person training sessions and minor administrative matters. The cost excludes travel and internet costs, which vary depending on the geographic location and internet service provider, respectively, and are borne directly by the participants. However, sponsorship is usually available to support some trainees.

During the course, trainees must have uninterrupted access to echocardiography imaging services at their locations of employment to build their skills. Numerous institutions countrywide have enthusiastically supported our HCWs trainees by allowing them to perform echocardiograms in their facilities. The program has also attracted support from the pharmaceutical and medical devices industry through grants, training equipment, and training facilities. In addition, prospective trainees across Africa have shown interest in participating in the program, which shows potential for scalability.

### Challenges, opportunities, and the impact of the KCS-ACC-TTE training program

Consistent access to echocardiography equipment by the trainees during training is not always possible due to high purchase and maintenance costs. This impairs trainees’ timely acquisition of psychomotor hands-on imaging skills and competency, which negatively impacts trainee confidence and autonomy in performing echocardiography (29). While initiatives like the National Managed Equipment Services (MES) program and proliferation of portable devices are improving access to imaging equipment, further investments are needed to ensure widespread availability, especially in rural areas (15,30).

The scarcity of expert faculty to train echocardiography poses a challenge (9,30). To overcome this, previous alumni of the KCS-ACC-TTE training program have been mentored into training by the faculty to ensure sustainability. Collaboration with other cardiology training institutions globally has expanded access to expert trainers, raising the quality of training. Online training platforms have helped overcome geographical and scheduling barriers, ensuring access to a broader portfolio of learning material. Reliable electricity supply and internet connectivity are available in more than 80% of Kenya’s health facilities, further enhancing access to online training platforms (18).

The estimated training cost of $1,400 may not be affordable in comparison to average annual salaries for HCWSs in Kenya which range from $ 4,000 to $ 25,000 depending on specialty, cadre, employer, and geographical area of practice (31). However, the program is currently considered sustainable owing to the KCS’s financial reserves and generous support from industry and the ACC. New avenues of funding will need to be explored for future sustainability.

In-depth training in advanced echocardiography modalities is not always possible due to specialized training infrastructure requirements and the trainees’ diverse clinical backgrounds. An opportunity to close this gap has emerged by conducting separate bi-annual advanced echocardiography workshops during the Kenya Cardiac Society Congress and the Africa STEMI Live conference targeted at suitable KCS-ACC-TTE course alumni to enhance targeted capacity building.

There is a lack of local cardiovascular imaging (CVI) data to guide training. As of 2022, nearly 70% of healthcare facilities in Kenya did not have an electronic health information system (EHIS) (18,32). Electronic data from CVI can facilitate the understanding of patterns of cardiac pathology, enable CV imaging research, and inform training priorities. Efforts to develop CVD registries to capture CVI data are underway to guide future training priorities and CVD care.

A TTE procedure in Kenya costs between $20 and $200, which is prohibitive for patients in LMICs, where out-of-pocket medical expenditure competes against other daily costs of living. Social health insurance programs only compensate for advanced cardiology procedures in select tertiary facilities. Cost barriers limit the number of patients who can access echocardiography, potentially restricting trainees’ exposure in secondary care facilities. Training efforts may increase the number of practitioners and lower the costs of echocardiography in the long term. However, continuous multi-level stakeholder involvement is required to advocate for sponsorships and sustainable social health programs to improve access. This will further improve the observed impact of the training program on CVD care countrywide.

**Figure 4:**
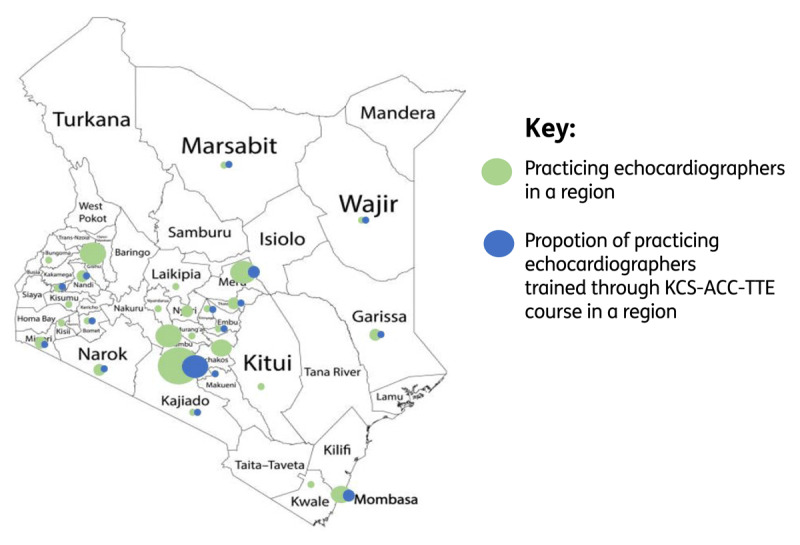
A proportion map highlighting the distribution of echocardiographers practicing in Kenya based on an exploratory survey done in June 2024 (Green circles). Circle size corresponds to the proportion of practicing echocardiographers in a region or county. Blue circles represent the proportion of practicing echocardiographers who have trained through the KCS-ACS-TTE course.

Despite these challenges, we are observing an impact of the KCS-ACC-TTE program in Kenya. An exploratory survey conducted in June 2024 assessed the reach of echocardiography services in Kenya among qualified trainees from various local TTE training programs. This data was presented during the 2024 KCS annual congress imaging workshop. Of the 70 respondents, 87% (n = 61) actively practiced echocardiography. Alumni of the KCS-ACC-TTE training program comprised 31% (n = 19) of those actively practicing. Overall, 72% (n = 44) of the participants practice within urban areas in Kenya, and 21% (n = 13) work in Nairobi. Most participants (48%) also work in level 5 and 6 hospitals. KCS-ACC-TTE course graduates work in at least 17 out of the 47 counties in Kenya, including marginalized counties such as Garissa, Wajir, Migori, Narok, Kajiado, and Tharaka Nithi as illustrated in Figure 4 below. On average, 1957 echocardiography procedures are performed weekly, or about 33 per survey participant per week. This exploratory survey presents self-reported data on the training, distribution, and workload of echocardiographers practicing in Kenya, thereby contributing foundational insight into a vital yet understudied component of service delivery in CVD care.

Further, anecdotal reports from KCS-ACC-TTE course graduates, trainees, and patients from these areas highlight the benefits of improved access to CVI. In Meru County five years ago, for example, patients traveled for more than 150 kilometers, taking at least three hours to Nyeri or Nairobi for a cardiology review and echocardiography. There is now one active alumnus of the KCS-ACC-TTE program in Meru and four more trainees from Meru, and the neighboring counties of Isiolo and Laikipia. Together with other trained practitioners, they form a network of HCWSs diagnosing and managing CVD, serving over three million people from this region. This has reduced the patient’s travel distance and time to less than 50 kilometers and less than an hour to access the service. This saves patients time and finances, facilitates early diagnosis, and enhances adherence to treatment and follow-up. Within Meru County, for example, some acute cardiac cases such as acute heart failure, myocardial infarction, pulmonary embolism with hemodynamic compromise, and pericardial effusion with tamponade have been diagnosed early and managed locally with guidance from cardiologists in tertiary centers with excellent outcomes.

Notably, increasing interest in the training program from prospective participants from other African countries shows potential for scalability beyond Kenya.

## Discussion

LMICs face critical barriers to effective CVD management, including inadequate human resources, limited healthcare infrastructure, high out-of-pocket healthcare costs, and gaps in guideline implementation. In Kenya, these challenges are further compounded by an uneven distribution of specialists, with the majority concentrated in urban private facilities, leaving rural populations underserved (19,20). Addressing these disparities necessitates innovative, sustainable solutions to enhance CVD care across all healthcare system levels.

The Kenya Cardiac Society-American College of Cardiology Transthoracic Echocardiography (KCS-ACC-TTE) training program represents a strategic capacity-building effort to bridge this gap. The program offers a blended learning model incorporating theoretical instruction, hands-on workshops, and ongoing mentorship, ensuring that trainees acquire and retain essential skills. By training non-cardiologists in echocardiography, the program aspires to enhance diagnostic capabilities of cardiac conditions in secondary and tertiary facilities, reducing reliance on the few cardiology experts in tertiary centers. This approach aligns with successful task-sharing models observed in other LMICs, such as Rwanda, where task-sharing had positive impacts on heart failure management and referral systems (10).

Comparisons may be drawn between the KCS-ACC-TTE training program to the Core Cardiology Training Symposium 4 (COCATS 4) introductory (level 1) TTE training done in the United States (33). Both the KCS-ACC-TTE training program and COCATS 4 Level 1 echocardiography training provide high-quality foundational education in TTE. Both programs emphasize structured learning, supervised hands-on training, and competency-based assessment to ensure proficiency. KCS-ACC-TTE course, like COCATS 4, integrates didactic instruction with practical experience, requiring trainees to perform and interpret echocardiograms under expert supervision. Both programs uphold rigorous training standards, preparing participants to assess cardiac anatomy, function, and pathology using echocardiography accurately. While COCATS 4 is designed for cardiology fellows within a training institution as a step to advanced cardiovascular imaging, the KCS-ACC-TTE program aims to train a broader group of healthcare workers in active clinical practice, offering echocardiography training that is adopted to resource-limited settings while upholding high training standards.

Despite its successes, the program faces systemic challenges, including limited access to equipment, a shortage of trainers, and financial barriers. Addressing these issues requires multi-stakeholder collaboration, policy interventions, and investments in infrastructure and training. The program’s blended learning model, combining online instruction with hands-on workshops, ensures skill acquisition and retention beyond geographic barriers, while mentorship fosters sustainability. Expanding health insurance coverage to include echocardiography and integrating training subsidies through government and private-sector partnerships could alleviate financial constraints for patients and trainees. Encouragingly, the KCS-ACC-TTE program has already garnered funding from partners, highlighting the potential for multi-stakeholder collaboration in sustaining and scaling such initiatives.

Preliminary data from a 2024 survey indicate that some KCS-ACC-TTE graduates are expanding echocardiography services to underserved regions, reducing patient travel distances and wait times. However, further research is needed to quantify these benefits, examining metrics such as impact on HCWs competency, reductions in misdiagnosis, improvements in clinical decision-making, indicators of accessibility, and patient satisfaction.

To enhance the program’s effectiveness, tiered training models are underway to cater to HCWSs at different levels of expertise, ensuring comprehensive skill development across the healthcare system. Establishing centralized cardiovascular imaging (CVI) registries would enable systematic data collection and track service provision, informing training priorities and policy. Standardizing training and certification across institutions would ensure competency and maintain high diagnostic standards and mitigate the ethical concerns of task sharing. Additionally, continually fostering collaborations—within Africa and globally—could facilitate knowledge exchange and bolster capacity-building efforts and scalability beyond Kenya.

## Conclusion

The KCS-ACC-TTE program exemplifies how targeted capacity-building initiatives can address critical gaps in CVD care. The program aspires to enhance diagnostic capabilities at secondary and tertiary facilities by equipping non-cardiologists with echocardiography skills, improving patient outcomes and alleviating pressure on specialized centers. However, sustaining and expanding these efforts requires strategic investments in training, equipment, and policy frameworks. Strengthening partnerships, exploring sustainable funding models, and leveraging technological advancements will be essential in ensuring long-term success. Ultimately, Kenya’s experience can serve as a blueprint for other LMICs seeking to enhance CVD care through innovative workforce development strategies.

## Data Accessibility Statement

Data obtained during the state of echocardiography survey in Kenya with the kind assistance of Health Data Acumen Limited is securely archived. It can be obtained for review by email on request through the first author.

## Additional File

The additional file for this article can be found as follows:

10.5334/gh.1437.s1 Supplementary Files. List of Figures.

## Abbreviations

ACC American College of Cardiology

BNP Brain Natriuretic Peptides

CHW Community Health Worker

COCATS Core Cardiology Training Symposium

CVD Cardiovascular Disease

CVI Cardiovascular Imaging

DALY Disability Adjusted Life Years

E FAST Extended Focused Abdominal Sonography for Trauma

EHIS Electronic Health Information System

FoCUS Focused Cardiac Ultrasound

HCWs Health Care Workers

KCS Kenya Cardiac Society

LMICs Low-to-Middle-Income Countries

MKTRH Mwai Kibaki Teaching and Referral Hospital

PoCUS Point-of-Care Ultrasound

TTE Transthoracic Echocardiogram

WHO World Health Organization
