# Supplementary material for: Cardiovascular Disease Care Beyond the Cardiologist: An Overview of the Rollout of Transthoracic Echocardiography Training and Services in Kenya
Source: Glob Heart. 2025 May 30;20(1):50. doi: 10.5334/gh.1437 (PMC12124244; doi:10.5334/gh.1437)
Supplement: Supplementary Files. — List of Figures. [file gh-20-1-1437-s1.pdf]

Cardiovascular Disease Care beyond the Cardiologist: Rollout of Trans-thoracic Echocardiography Training and Services in Kenya

Supplementary files: List of Figures

Authors: Muriuki Daniel M.[1], Nguchu Karimi H.[2], Ahmed Hassan[3], Foster Mike[4], Goldberg, Lee R.[5] Ambrose Marietta S.[6] Samia Bernard[7]

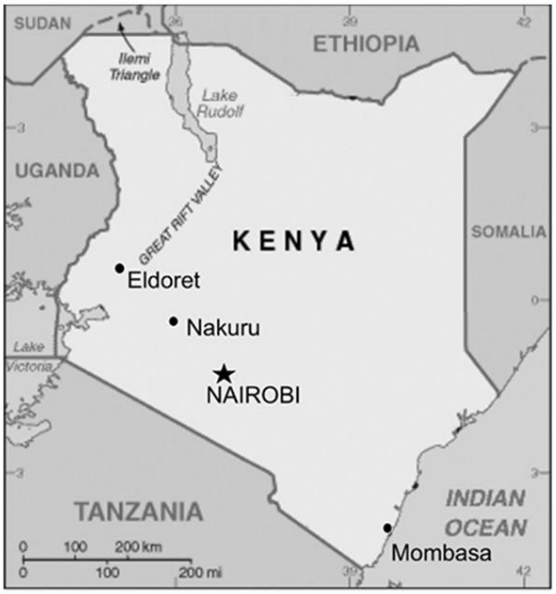

Figure 1: A map of Kenya showing the geographic localization of cardiology experts in 2009. No current data mapping their geographic distribution is available. (Courtesy, Binnanay et al *(16)*)

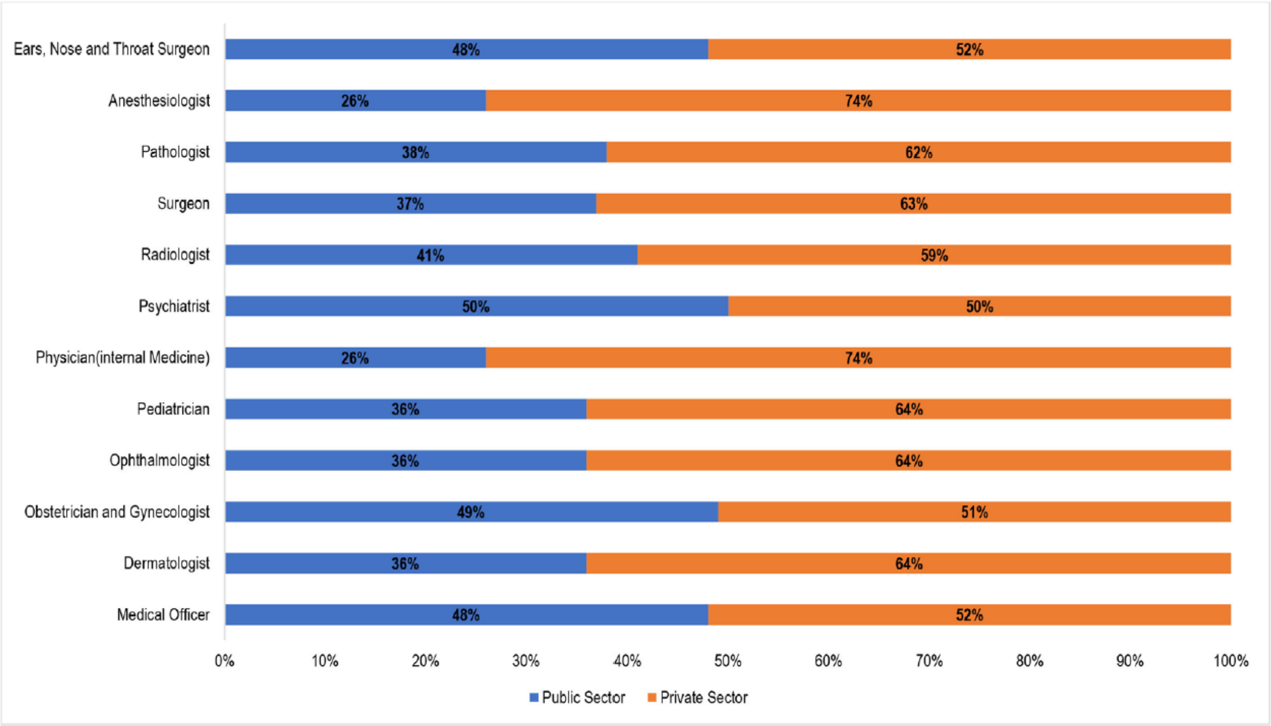

Figure 2: Distribution of selected HCW in Kenya in the private vs public sector. Courtesy Okoroafor et al. *(17)*

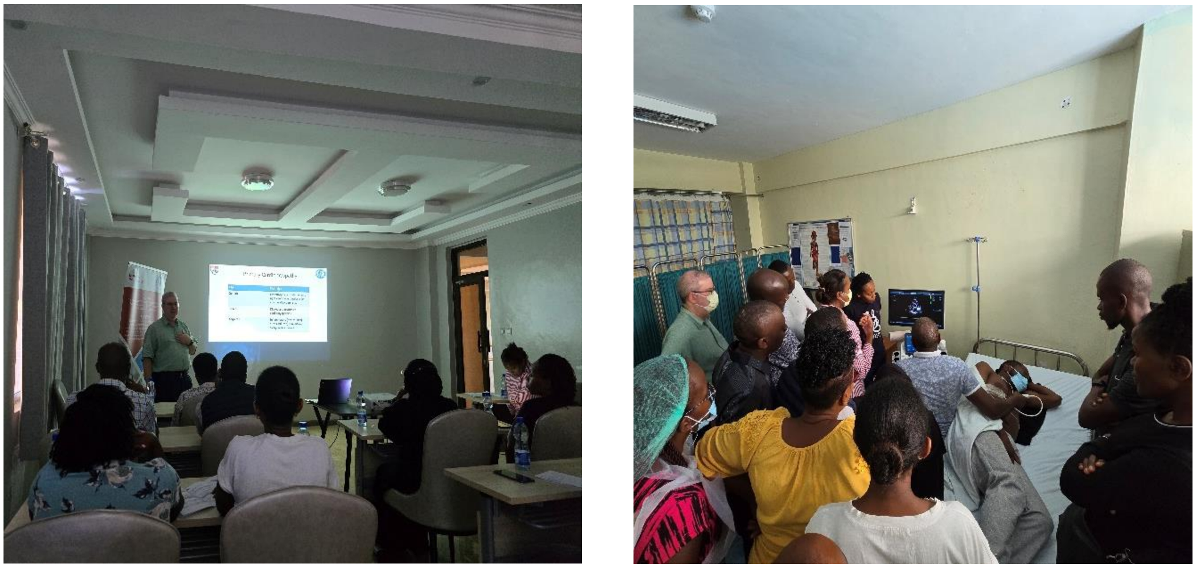

Figure 3: In-person (left) and hands-on training (right) sessions in January 2024 with KCS-ACC faculty. Photo: Mwai Kibaki Teaching and Referral Hospital (MKTRH). reproduced with permission from MKTRH

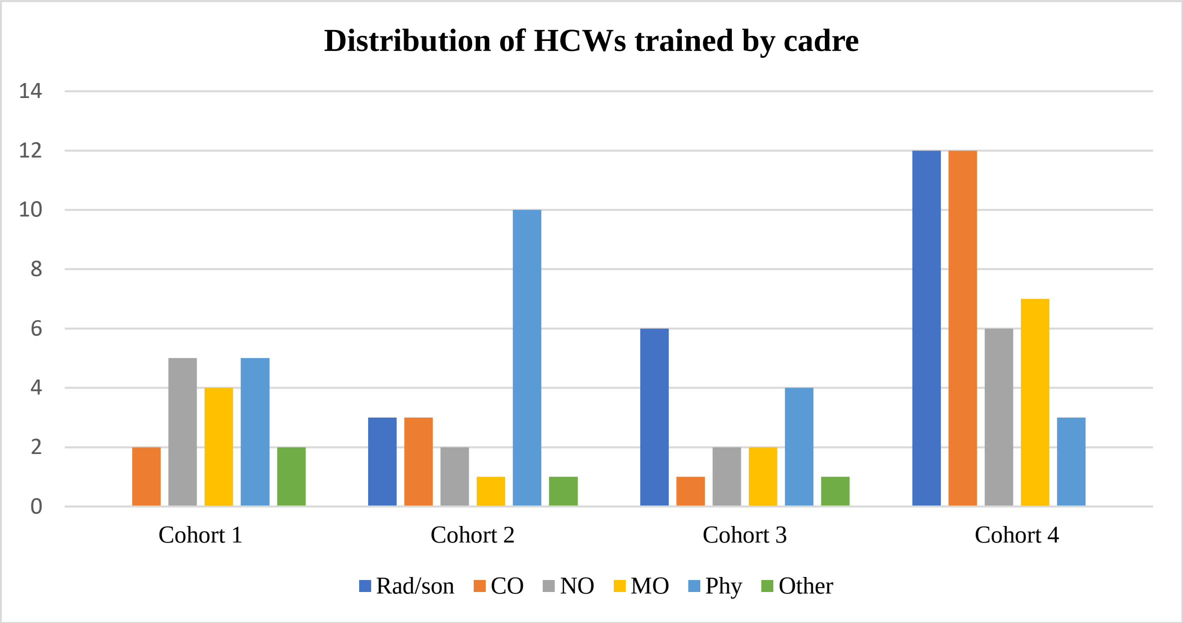

Figure 1: Distribution of HCWs by cadre across the four training cohorts.

Key: Rad/son- Radiographer/sonographer, **CO**- Clinical Officer, **NO**- Nursing Officer, **MO**- Medical Officer, **Phy**- Physician

*Other: Two anesthesiologists, one nephrologist, one cardiothoracic surgeon

Code

Echo_Practice_Kenya

Olushina Ale - Health Data Acumen (HDA) & Holo Global Health Research Institute
2024-07-23

1 Type of Trainees in Echocardiography

Gender repartion by cadre on data collected on the state of echocadiography in Kenya in July 2024

		Gender		
**Cadre**		**Female**	**Male**	**Total**
Clinical Officer	Count	7 (10.0%)	18 (25.7%)	25 (35.7%)
	Mar. pct (1)	25.0% ; 28.0%	42.9% ; 72.0%	
Medical Officer	Count	1 (1.4%)	1 (1.4%)	2 (2.9%)
	Mar. pct	3.6% ; 50.0%	2.4% ; 50.0%	
Nursing Officer	Count	3 (4.3%)	2 (2.9%)	5 (7.1%)
	Mar. pct	10.7% ; 60.0%	4.8% ; 40.0%	
Other	Count	6 (8.6%)	7 (10.0%)	13 (18.6%)
	Mar. pct	21.4% ; 46.2%	16.7% ; 53.8%	
Radio-sonographer	Count	4 (5.7%)	7 (10.0%)	11 (15.7%)
	Mar. pct	14.3% ; 36.4%	16.7% ; 63.6%	
Specialist Cardiologist	Count	2 (2.9%)	2 (2.9%)	4 (5.7%)
	Mar. pct	7.1% ; 50.0%	4.8% ; 50.0%	
Specialist Physician	Count	5 (7.1%)	5 (7.1%)	10 (14.3%)
	Mar. pct	17.9% ; 50.0%	11.9% ; 50.0%	
Total	Count	28 (40.0%)	42 (60.0%)	70 (100.0%)

(1) Columns and rows percentages

```
The 'Other' category in the 'Cadre' column includes the following specified roles:
 -  NA
-  Neurophysiology Technologist
-  Cardiac technician
-  Cardiac Sonographer
-  Registrar
-  Cardiac Technologist
-  Cardiovascular technologist
-  Clinical officers specialist -cardiology
-  Technologist
-  Specialist clinical officer-cardiology
```

```
-  Echocardiography Technician
-  Cardiac nurse/sonography
-  Clinical cardiologist
```

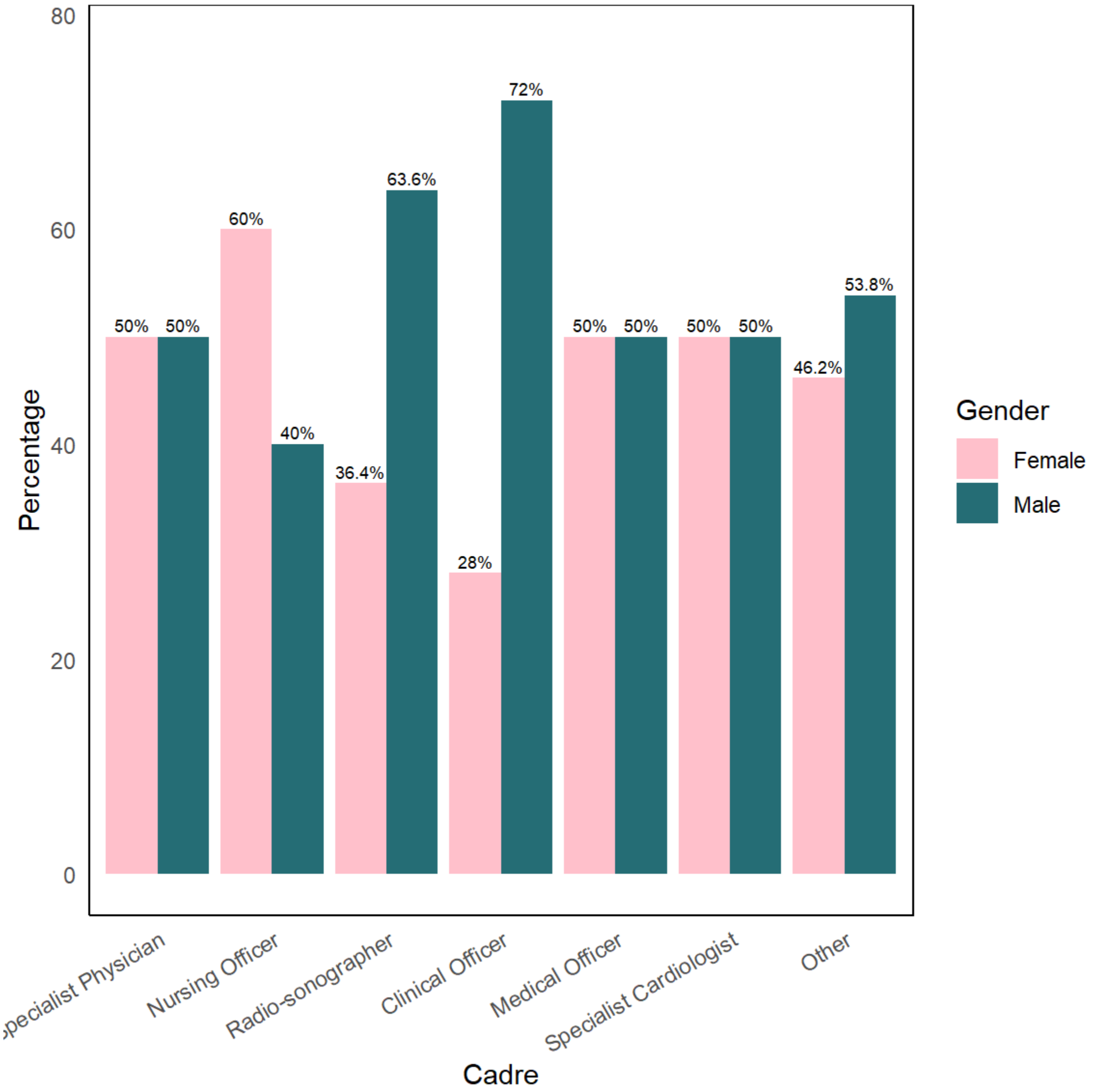

Cadre	Mean_age	Median_age
Specialist Physician	39.7	37.5
Nursing Officer	35.8	37.0
Radio-sonographer	32.0	30.0
Clinical Officer	33.1	34.0

Cadre	Mean_age	Median_age
Medical Officer	32.0	32.0
Specialist Cardiologist	46.0	42.5
Other	34.9	34.0

2 Place were the respondents are trained

Summary of Training Places for Cardiac Diagnostic Services

Training Places	Count	Percentage
Echo4Life	35	50.00
KCS-ACC	19	27.14
Other	10	14.29
Karen Hospital	8	11.43
KMTC	3	4.29

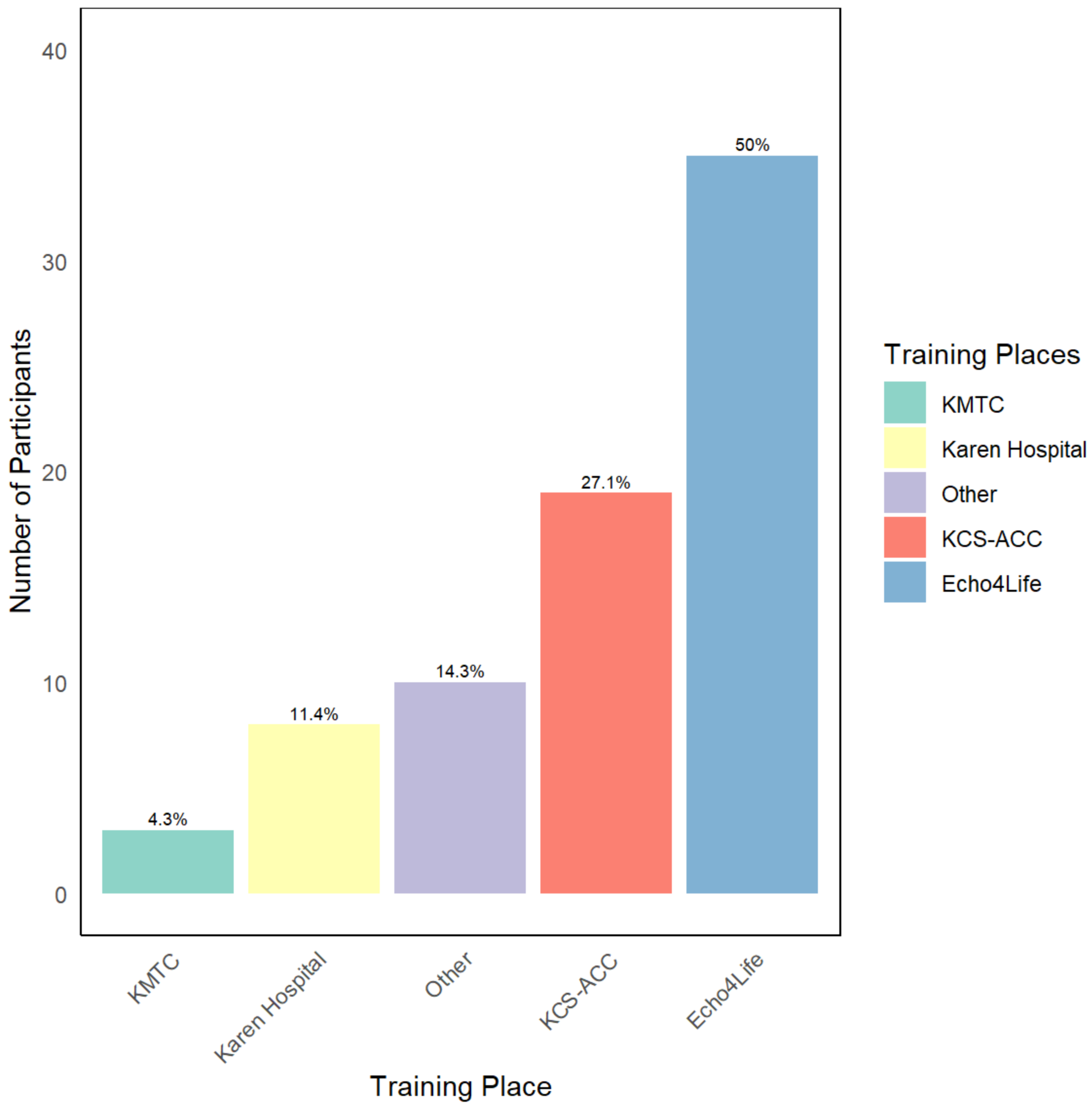


```
The 'Other' category in the 'Where trained' column includes the following specified places:
 -  Medical University of Vienna
-  Manipal Academy of Higher Education
-  UK
-  123 sonography
-  Mtrh
-  MTRH COLLEGE OF MEDICINE
-  AKUHN
-  India
-  Madras Medical Mission Hospital India
```

3 Of those trained, how many are actively practicing?

```
Among the 70 participants who have received training, 61 ( 87.1 %) are currently practicing.
```

4 Where are they practicing? Which county? Urban or rural? Which type of practice?

Summary of Participants by County

County of current practice (state)	Count	Percentage
Nairobi	13	21.31
Kenya	8	13.11
Meru	3	4.92
Kiambu	2	3.28
Mombasa	2	3.28
Uasin Gishu	2	3.28
Bomet	1	1.64
Bungoma	1	1.64
Embu	1	1.64
Garissa	1	1.64
KIAMBU	1	1.64
Kajiado	1	1.64
Kiambu County	1	1.64
Kisii	1	1.64
Kisumu	1	1.64
Kitui	1	1.64
Kwale County	1	1.64
Laikipia	1	1.64
Machakos	1	1.64
Migori	1	1.64
Mimbasa	1	1.64
Muranga	1	1.64
NAIROBI	1	1.64

Summary of Participants by County

County of current practice (state)	Count	Percentage
Nairobi county	1	1.64
Nairobi i	1	1.64
Nakuru county	1	1.64
Nandi	1	1.64
Narok	1	1.64
Narok county refferal hospital	1	1.64
Nyeri	1	1.64
Tharaka nithi	1	1.64
Tharaka-Nithi	1	1.64
Uasingishu	1	1.64
United Kingdom	1	1.64
Uwezo healthcare	1	1.64
meru	1	1.64
nyeri	1	1.64

Summary of Participants by Setting

Setting of practice…38	Count	Percentage
Urban	44	72.13
Rural	17	27.87

Summary of Participants by Type of Practice

Type of practice	Count	Percentage
Level 5 or 6 hospital (tertiary/referral center)	29	47.54
Diagnostic center	19	31.15
Level 4 hospital (secondary facility)	9	14.75
Other out-patient	4	6.56

```
Among the 61 participants currently practicing, the majority are in Nairobi with 13 ( 21.3 %),
practicing in Urban with 44 ( 72.1 %), and in Level 5 or 6 hospital (tertiary/referral center)
with 29 ( 47.5 %).
```

5 What other cardiac diagnostic services are offered where they practice?

Summary of Cardiac Diagnostic Services Offered in Practicing Facilities

Procedures	Count	Percentage
Resting ECG	56	91.80
Resting echocardiogram	40	65.57
Ambulatory ECG (Holter)	22	36.07
Stress ECG	18	29.51
Cardiac Catheterization lab	15	24.59
Stress echocardiogram- Contrast echocardiogram	13	21.31
Cardiac CT	11	18.03
Other	8	13.11
Cardiac MRI	7	11.48

6 How many procedures do they do per week?

```
Among the 61 participants currently practicing, a total of 1957 echocardiograms are performed
per week, averaging 32.08 procedures per week per participant.
```

7 What conditions do they commonly encounter during practice?

```
# A tibble: 9 × 2
  Condition                          Average_Frequency
  <chr>                                          <dbl>
1 Hypertensive heart disease                      4.64
2 Pulmonary hypertension                          3.95
3 Right heart failure                             3.80
4 Normal                                          3.67
5 Valvular heart disease                          3.54
6 Ischemic dilated cardiomyopathy                 3.48
7 Non-ischemic dilated cardiomyopathy             3.39
8 Pericardial disease                             2.92
9 Restrictive cardiomyopathy                      2.52
[1] "The conditions most commonly encountered during practice are: Hypertensive heart disease
."
```

×

- 1 **Type of Trainees in Echocardiography**
- 2 **Place were the respondents are trained**
- 3 **Of those trained, how many are actively practicing?**
- 4 **Where are they practicing? Which county? Urban or rural? Which type of practice?**
- 5 **What other cardiac diagnostic services are offered where they practice?**
- 6 **How many procedures do they do per week?**
- 7 **What conditions do they commonly encounter during practice?**
